# A Comparison of Two Anastomotic Techniques in the Jejunum of the Goat

**DOI:** 10.4061/2010/139610

**Published:** 2010-11-01

**Authors:** H. A. Al-Timmemi, Karim Al-Jashamy, Mohammed S. Dauod

**Affiliations:** ^1^Faculty of Veterinary Medicine, Universiti Putra Malaysia, Selangor 43400, Malaysia; ^2^Medical School, SEGi University College, 47810 Petaling Jaya, Selangor, Malaysia; ^3^Faculty of Veterinary Medicine, Baghdad University, 1963 Al-aameria, Baghdad, Iraq

## Abstract

This study was carried out to test two different anastomotic techniques to identify advantages and disadvantages of each technique in goats. All animals were under local infiltration anaesthesia. A five-cm length of jejunum was resected from the first part of the jejunum and end to end anastomosis using 3-0 Polygalactin-910 with one row of sero-submoucosal interrupted sutures (SSIS) group, and one row of horizontal mattress interrupted sutures (HMIS) group. Two animals from each group were euthanized on the 4th, 14th and 21st postoperative days. A 7-cm segment of jejunum including the anastomosed area was resected from each animal. There was no significant adhesion between anastomosis area and surrounded tissues observed in SSIS animals, while there was significant adhesion between anastomosis area and surrounded tissues which were observed in HMIS animals. Stenosis degree was lower in the SSIS than the HMIS group. The bursting pressure was higher in the SSIS than the HMIS group. Macroscopic evaluation indicated that the anastomotic line mucosa was abridged better with less local edema in the SSIS group. Histological evaluation in the SSIS group showed almost all parameters such as epithelial recovery and repair of submucosal-mucosal layer demonstrated better healing compared to the HMIS group.

## 1. Introduction

Intestinal anastomosis is a basic procedure in gastrointestinal surgery. Manual intestinal anastomosis has been practiced for centuries, and it is still considered an option among the preferred types of anastomotic techniques. There is still interest in research on intestinal anastomosis because failed anastomosis is associated with high morbidity and mortality [1]. Although manual intestinal anastomosis has been practiced for centuries, it is still considered as an option among the preferred types of anastomotic techniques [2, 3]. Anastomotic technique is an important part of successful anastomosis healing [4]. The objective of this study was to test two different anastomotic techniques in order to identify the advantages and disadvantages of each in goats.

## 2. Materials and Methods

### 2.1. Experimental Animals

Twelve clinically healthy adult (2–2.5 years) local mixed breed goats (20.0–25.0 kg) were purchased from a local commercial farm. The experimental protocols, animal ethics, and animal welfare were approved by the Animal Care and Use Committee (VETBAG/3.2.06/surg 40), Faculty of Veterinary Medicine, Baghdad University, Iraq. Prior to the commencement of the experiment, goats were kept for an acclimatization period of three weeks during which time they were fed concentrate fodder and were able to drink water *ad libitum. *They were dewormed using a single subcutaneous dose of ivermectin 200 *μ*g/kg (Ivomec Drench, USA). The animals were divided randomly into two groups, the first group received sero-submoucosal interrupted sutures (SSIS) and the second group received horizontal mattress interrupted sutures (HMIS).

### 2.2. Preoperative Preparation and Laparotomy

Food and water were withheld for 12 hrs and pen-strep 20 mg/kg IM (Norbrook, UK) was given half an hour before the surgery prophylactically. The animals were placed in left lateral recumbency on the opposite side of the surgical operation. The right flank of each animal was clipped, shaved, and prepared aseptically. Surgeries were performed under sedation with lidocaine 0.05 mg/kg IM (Ceva, Germany) and regional local anaesthesia of lidocaine 2% 1 mL/cm^2^ (Panther, UK) [5]. A 10-cm incision through the skin, subcutaneous fascia, was performed. The external abdominal oblique muscle, internal abdominal oblique, and transverse abdominal muscles were separated by blunt dissection, the peritoneum opened, and the jejunum exteriorised. A 5-cm segment was resected from the proximal jejunum. End-to-end anastomosis was performed using 3–0 Polygalactin-910 (Vicryl; Ethicon, UK) with one row of serosubmucosal interrupted sutures in SSIS animals and one row of horizontal mattress interrupted sutures in HMIS animals. After the anastomosis, the jejunum was returned to its normal place after being washed with physiological saline (0.9% NaCl). The peritoneum and muscular layers were sutured by simple continuous pattern using 1 : 1200 diluted polyglycolic acid and the skin then sutured with 0 silk in a simple interrupted suture pattern.

All animals were provided dextrose 5% throughout the operative surgery, and pen-strep 20 mg/kg IM was given for 4th postoperative day (POD). The animals were under human care observations for medication and distress helping. On the first postoperation day, the animals were provided with only a little water. On the second and third days, they fed on green grass, and then on normal fodder. Two animals from each group were euthanized on the 4th, 14th, and 21st PODs using intravenously injected sodium pentobarbital 200 mg/mL (0.5 mL/kg, BW).

### 2.3. Pathology

The gross pathology of the skin, abdominal wall wounds, and the abdomen was opened to evaluate the healing of the anastomosis and adhesion formation. Intra-abdominal adhesions were assessed and graded using a standard scale according to the following criteria [6]. O point: No adhesion; 1 point: slight adhesion; 2 points: mild adhesion; 3 points: Severe adhesion. After identification of the anastomosed area, a 7-cm segment of jejunum with the anastomotic site was resected, transected longitudinally, and rinsed with saline to remove intestinal contents. Specimens were fixed in 10% formalin for histopathological examination.

### 2.4. Histopathological Examination

Tissue specimens obtained from the anastomotic site were embedded into paraffin blocks following routine histochemical procedures. Five micron thick sections were stained with Hematoxylin-Eosin and examined under light microscope. Degree of wound healing at the line of anastomosis was graded on a scale from 1 to 5: Grade 1: fibrinopurulent exudate, grade 2: granulation tissue less than 25%, grade 3: granulation tissue between 25–75%, grade 4: granulation tissue more than 75% or intestinal epithelial cells from intact intestinal glands and short villi less than 25%, grade 5: intestinal epithelial cells from intact intestinal glands and short villi and microvilli more than 25% [7].

### 2.5. Stenosis Degree

One end of the jejunum segment was closed and filled with 25% Barium sulfate contrast medium for X-ray examination and the second end then closed also. The specimens were examined by X-ray using MAS 3, Kv 50 (Shimadza, Japan) to measure the intestinal diameter and stenosis in the area of anastomosis within 2 cm before and after the anastomotic line using the following formula:

(1) $$\text{Narrowing}=100\left(1-\frac{2A}{B+C}\right).$$

*A* is the intestinal diameter in the anastomosis area. *B* and *C* are the intestinal diameters 2 cm before and after the anastomosis site [8].

### 2.6. Bursting Pressure

To measure the intestinal bursting pressure, one end of the jejunum segment was closed, another end linked to an air pump, and the specimen was put into water and filled with air to identify leakage or rupture in the anastomosis site. The air pump scale reading was recorded at leakage or rupture, and this represented the bursting pressure (mmHg) [9]. Data obtained from the study group were expressed as mean ± standard deviation and subjected to statistical analysis using Statistical Package for the Social Sciences (Version 11.0 for windows; SPSS, USA). The sample size at each time point is small; it would be helpful to provide more details regarding the statistical analysis, for example, which statistical test was used for the different statistical comparisons reported in the paper.

## 3. Results

### 3.1. Clinical Examination

Wound inspection and clinical examination were performed as part of daily followup. Operative wounds healed well and skin sutures were removed on the 8th POD in the animals which were euthanized on the 14th and 21st POD. All animals showed normal clinical signs and good appetite for water and food until the end of experiment.

### 3.2. Stenosis Degree

**Figure 1:**
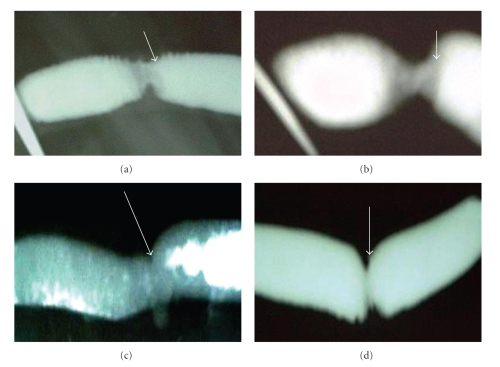
X-ray photographs of experimental intestinal anastomosis in goat showing different stenosis degrees (arrows). (a) Sero-submoucosal interrupted suturing (SSIS) group on 4th postoperative day (POD). (b) HMIS group on 4th POD. (c) SSIS group on 21st POD. (d) HMIS group on 21st POD.

There was significant intestinal stenosis degree (*P* < .01) in both SSIS and HMIS animals euthanized on the 4th POD (Figures 1(a) and 1(b)). No significant intestinal stenosis (*P* < .05) was observed in the animals euthanized on the 14th and 21st PODs from the SSIS group (Figure 1(c)) while significant intestinal stenosis was observed in the HMIS groups at the same time points (*P* < .05) (Figure 1(d)).  Table 1 summarizes the degree of stenosis.

### 3.3. Bursting Pressure

There were significant differences in bursting pressures in both groups but the SSIS group showed a higher bursting pressure than the HMIS group on the 21st POD (*P* < .01). In the Table 2, the SSIS pressure is lower than the HMIS pressure on day 8, but higher on days 14 and 21.

### 3.4. Pathology

#### 3.4.1. Macroscopic Examinations

**Figure 2:**
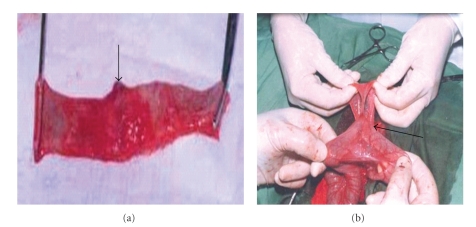
Photographs of experimental intestinal anastomosis in goat. (a) The anastomosis site (arrow) in the SSIS group on 21st POD. (b) Adhesion between intestinal anastomosis site and omentum (arrow) in horizontal mattress interrupted suturing (HMIS) group on 21st POD.

No adhesions between the anastomotic line, peritoneum, and abdominal wall were observed at postmortem in the SSIS group (Figure 2(a)) except in 2 animals in which slight adhesion between the anastomotic line, abdominal wall, and peritoneum was seen on the 4th and 14th PODs. The animals in the HMIS group showed different degrees of adhesions between the anastomotic line and peritoneum, omentum and intestine (Figure 2(b), Table 3).

#### 3.4.2. Histopathological Findings

**Figure 3:**
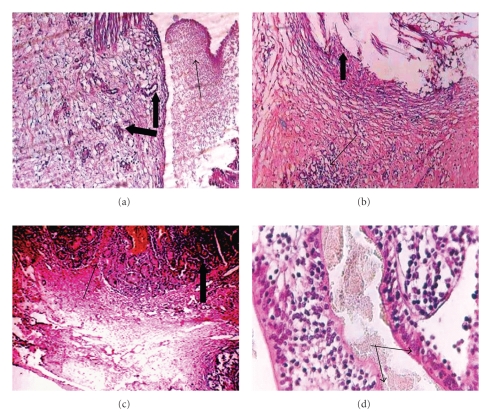
Histopathology photographs of experimental intestinal anastomosis in goat, SSIS group. (a) Organized thrombus in the anastomosis site on 4th POD (thin arrow) and new blood vessels (thick arrow), H&E stain, ×10. (b) On 14th POD: granulation tissue infiltrated with lymphocytes in the serosa and submucosa, new blood vessels (thin arrows), and epithelial cells (thick arrow), H&E stain, ×10. On 21st POD, (c) anastomotic line completely filled with scar tissue (thin arrow), scant fibroblasts, lymphocytes, epithelial cells, intestinal glands, and crypts of Lieberkühn (thick arrow), H&E ×20. (d) Long villi and microvilli (arrow) lined the intestinal mucosa, H&E stain, ×40.

The histopathological findings of the intestinal specimens from euthanized animals on the 4th POD in the SSIS group were characterized by hematoma, granulation tissue, and infiltration with inflammatory cells (predominately neutrophils and macrophages) in the anastomotic line. Granulation tissue had developed in the serosa and submucosa with new blood vessels as a grade 2. Crypts of Lieberkühn were also seen in the mucosa (Figure 3(a)).

On the 14th POD, the sutures were absorbed leaving some vacuoles surrounded by granulation tissue infiltrated with inflammatory cells (predominately lymphocytes) and intestinal epithelial cells in the anastomotic line which led to an increase in the thickness of the mucosal layer as a grade 3 (Figure 3(b)). 

On the 21st POD, the anastomotic line was completely filled with scar tissue that showed as a dense connective tissue, scanty fibroblasts, plenty of new blood vessels, and crypts of Lieberkühn in the regeneration area. New intestinal villi and microvilli were seen in the regenerated area that was infiltrated with scant lymphocytes and epithelial cells as a grade 5 (Figures 3(c) and 3(d)).

**Figure 4:**
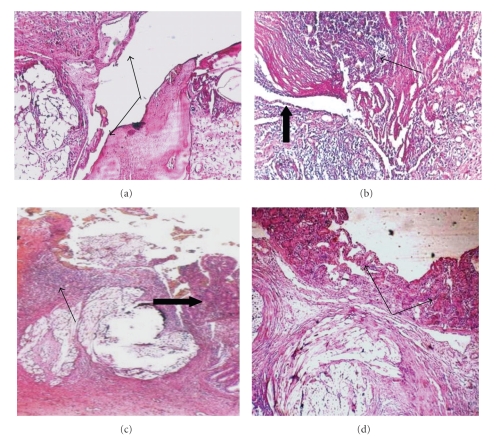
Histopathology photographs of experimental intestinal anastomosis in goat, HMIS group. (a) On 4th POD: degeneration, necrosis, fibroblasts, infiltration of neutrophils, and necrosis of intestinal villi, (arrows), H&E ×10. (b and c) On 14th POD: connective tissue, wound edges infiltrated with macrophages and lymphocytes (thin arrow), and few intestinal epithelial cell infiltrated from intact intestinal glands (thick arrow), H&E ×20. (d) On 21st POD: organization of connective tissue, fibroblasts in an anastomosis area and short villi lined the intestinal mucosal surface (arrows), H&E stain, ×20.

The histopathology slides of the HMIS group on the 4th POD showed degeneration, edema, plenty of fibroblasts, infiltration of neutrophils, necrosis of intestinal gland epithelial cells, necrosis of intestinal villi, unabsorbed stitches surrounded by granulation tissue infiltrated with inflammatory cells, and lack of wound edge alignment as a grade 2 (Figure 4(a)). 

On the 14th POD, there was a granulation tissue connecting the wound edges which was infiltrated with macrophages, lymphocytes, and scanty intestinal epithelial cells from intact intestinal glands as a grade 3 (Figures 4(b) and 4(c)). On the 21st POD, organized connective tissue and fibroblasts in the anasomostic area with short villi were seen as a grade 4 (Figure 4(d)).

## 4. Discussion

There was a significant difference in the percentage reduction of lumen diameters between the two techniques; there was evidence of continuous stenosis and obstruction observations from the anastomotic technique in the HMIS group at 21st POD. Histopathologically, the inflammatory response and fibrosis were minimal with the SSIS technique with grade 5, while there was increased fibrosis and suture tract inflammation in the HMIS technique which only showed grade 4. Anastomosis quality was assessed by the following parameters: endoluminal and extraluminal macroscopic appearance of the anastomotic line, perianastomotic adhesion density, the bursting pressure of the anastomoses, and histological changes during the healing phases. There have been numerous clinical and experimental studies on surgical techniques and the healing process of intestinal anastomosis [1–3, 10]. Traditionally, surgical sutures have been used to perform manual intestinal anastomoses which are mainly made by staplers, and training of surgeons in manual anastomosing is still very much needed. There are different preferences among surgeons regarding the use of surgical techniques for creating intestinal anastomoses [3, 11]. 

The present study focused on the healing of intestinal anastomosis on the 4th, 14th, and 21st PODs; findings were characterized by restoration of the matrix accumulation and the strength of anastomosis. Endoluminally, the macroscopic appearance of the anastomotic line was quite similar in specimens of both groups on the 4th POD. However, on the 14th and 21st PODs, the anastomotic line was found to be less edematous with better mucosal coverage in the SSIS group compared to the HMIS group. This was probably due to better apposition of intestinal layers, less narrowing of the lumen, and a smaller amount of strangulated tissue. Another aspect analyzed by this study was the density of intraperitoneal adhesions. The density of the intraperitoneal adhesions was quantified according to Singer et al. [12]. Significant differences were found in the intraperitoneal adhesion in HMIS (grade 3) compared to SSIS (grade 2). Degree of adhesion was influenced by the anastomosis technique and the time after surgery. Previous studies have shown that adhesions at an anastomosis can be caused by the contamination of the peritoneal area, sutures (foreign material), and ischemic changes of the intestine in the anastomotic region [13–15]. A significant increase of the biomechanical parameters of the anastomotic line between the 4th, 14th, and 21st PODs has also been demonstrated in other studies [8, 16], which found significant increases of vascular perfusion in areas of the anastomosis 3 days after surgery. These studies confirmed the importance of angiogenesis and other concomitant changes in increasing the strength of anastomosis after the first 3 days.

Cellular and architectural parameters of anastomotic healing were evaluated. Histopathological changes, such as presence of necrosis, polymorphonuclear infiltration, edema, epithelial recovery, and repair of submucosal-muscular layer have shown that the technique in SSIS was closer to “ideal healing” than the HMIS technique. In conclusion, the results of the present study indicate that the SSIS technique shows better biomechanical and histological parameters on the 14th and the 21st PODs compared to the HMIS technique.

## Figures and Tables

**Table 1:** The stenosis degree (%) in the serosubmucosal interrupted sutures (SSIS) and horizontal mattress interrupted sutures (HMIS) groups.

Groups/POD*	4th day Mean ± SD	14th day Mean ± SD	21st day Mean ± SD
SSIS	65.24 ± 3.9	46.87 ± 3.6	27.36 ± 1.05
HMIS	69.67 ± 3.5	49.7 ± 4.5	42.08 ± 1.4**

*POD: postoperative day, **Stenosis degree (%).

**Table 2:** The bursting pressure mmHg in the SSIS and HMIS groups.

Groups/POD*	4th day Mean ± SD	14th day Mean ± SD	21st day Mean± SD
SSIS	90.5 ± 0.5	146 ± 1.01	152.5 ± 2.5**
HMIS	91.5 ± 1.51	137.5 ± 2.5	142.5 ± 2.5

*POD: postoperative day, **Bursting pressure mmHg.

**Table 3:** The number of animals which got adhesion, adhesive degrees, and sites in the SSIS and HMIS groups.

Group	Number of animals	POD*	Degree of adhesive	Adhesive site
SSIS	1	4th	Slight adhesion	abdominal wall
	1	14th	Slight adhesion	peritoneum

HMIS	1	14th	Severe adhesion	Peritoneum, omentum and intestine
	2	21st	Severe adhesion	
	1	4th	Mild adhesion	omentum and intestine
	2	14th	Slight adhesion	Intestine

*POD: postoperative day.
